# Inhibiting acid‐sensing ion channel exerts neuroprotective effects in experimental epilepsy via suppressing ferroptosis

**DOI:** 10.1111/cns.14596

**Published:** 2024-02-15

**Authors:** Xiaorui Shi, Ru Liu, Yingting Wang, Tingting Yu, Kai Zhang, Chao Zhang, Yuyu Gu, Limin Zhang, Jianping Wu, Qun Wang, Fei Zhu

**Affiliations:** ^1^ Department of Neurology, Beijing Tiantan Hospital Capital Medical University Beijing China; ^2^ China National Clinical Research Center for Neurological Diseases Beijing China; ^3^ Advanced Innovation Center for Human Brain Protection Capital Medical University Beijing China; ^4^ Department of Neurosurgery, Beijing Tiantan Hospital Capital Medical University Beijing China; ^5^ Center of Epilepsy, Beijing Institute of Brain Disorders, Collaborative Innovation Center for Brain Disorders Capital Medical University Beijing China

**Keywords:** acid‐sensing ion channel, disease‐modifying therapies, drug‐resistant epilepsy, ferroptosis, neuroprotection

## Abstract

**Background:**

Epilepsy is a chronic neurological disease characterized by repeated and unprovoked epileptic seizures. Developing disease‐modifying therapies (DMTs) has become important in epilepsy studies. Notably, focusing on iron metabolism and ferroptosis might be a strategy of DMTs for epilepsy. Blocking the acid‐sensing ion channel 1a (ASIC1a) has been reported to protect the brain from ischemic injury by reducing the toxicity of [Ca^2+^]_i_. However, whether inhibiting ASIC1a could exert neuroprotective effects and become a novel target for DMTs, such as rescuing the ferroptosis following epilepsy, remains unknown.

**Methods:**

In our study, we explored the changes in ferroptosis‐related indices, including glutathione peroxidase (GPx) enzyme activity and levels of glutathione (GSH), iron accumulation, lipid degradation products‐malonaldehyde (MDA) and 4‐hydroxynonenal (4‐HNE) by collecting peripheral blood samples from adult patients with epilepsy. Meanwhile, we observed alterations in ASIC1a protein expression and mitochondrial microstructure in the epileptogenic foci of patients with drug‐resistant epilepsy. Next, we accessed the expression and function changes of ASIC1a and measured the ferroptosis‐related indices in the in vitro 0‐Mg^2+^ model of epilepsy with primary cultured neurons. Subsequently, we examined whether blocking ASIC1a could play a neuroprotective role by inhibiting ferroptosis in epileptic neurons.

**Results:**

Our study first reported significant changes in ferroptosis‐related indices, including reduced GPx enzyme activity, decreased levels of GSH, iron accumulation, elevated MDA and 4‐HNE, and representative mitochondrial crinkling in adult patients with epilepsy, especially in epileptogenic foci. Furthermore, we found that inhibiting ASIC1a could produce an inhibitory effect similar to ferroptosis inhibitor Fer‐1, alleviate oxidative stress response, and decrease [Ca^2+^]_i_ overload by inhibiting the overexpressed ASIC1a in the in vitro epilepsy model induced by 0‐Mg^2+^.

**Conclusion:**

Inhibiting ASIC1a has potent neuroprotective effects via alleviating [Ca^2+^]_i_ overload and regulating ferroptosis on the models of epilepsy and may act as a promising intervention in DMTs.

## Graphical abstract

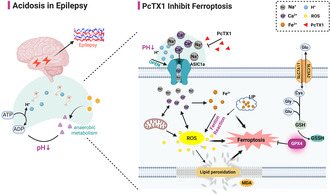

## 1 INTRODUCTION

Epilepsy is one of the most prevalent central nervous system disorders, which features recurrent seizures caused by sudden hypersynchronous neuron discharges.^1^ Although the first‐line treatments for epilepsy are anti‐seizure medications (ASMs), about 30% of patients with epilepsy fail to benefit from seizure control.^2^, ^3^ The traditional ASMs mainly target remodeling the balance of excitation and inhibition, including regulating ligand‐gated glutamate receptors, enhancing γ‐aminobutyric acid (GABA) function, etc.^4^ However, epilepsy is a chronic progressive disease with cell damage, triggering the inflammation response and recapitulation of development.^5^ In 2002, Löscher et al.^6^ proposed that the development of disease‐modifying therapies (DMTs) is one of the important future goals for epilepsy treatment. However, there is currently no drug available that can ameliorate the course of epilepsies and related comorbidities. Therefore, developing effective and safe DMTs is a high priority in epilepsy research and care.^7^

Acidosis, the process of tissue pH reductions induced by accumulating lactic acid following the release of H^+^ from ATP hydrolysis, has been found in the epileptogenic foci of patients with drug‐resistant epilepsy (DRE).^8^, ^9^, ^10^ Notably, acid‐sensing ion channels (ASICs) are a family of ion channels mainly activated by H^+^. They are expressed throughout the central and peripheral nervous systems, including the brain, spinal cord, and sensory ganglia.^11^ So far, there are mainly four members with several splice variants (ASIC1a, ASIC1b, ASIC2a, ASIC2b, ASIC3, and ASIC4) encoded by four different genes (Asic1, Asic2, Asic3, and Asic4) in mammals.^11^, ^12^, ^13^ Among them, ASIC1a is a subtype located on the neurons in central in the central nervous system, in which they are sensitive to extracellular pH alternations below 7. When H^+^ binds to the extracellular domain of ASIC1a, the channel is activated, and Na^+^ and Ca^2+^ pass through the pore into the cell, leading to membrane depolarization and downstream cellular effects.^14^, ^15^, ^16^ In a previous study, we detected six tag single‐nucleotide polymorphisms of the ASIC1a encoding gene in 560 patients with temporal lobe epilepsy (TLE) and 401 healthy controls. Notably, we found that an ASC1a variant allele (rs844347: A>C) was significantly associated with TLE.^17^ However, this study is limited to direct and more powerful evidence based on brain tissue.

Interestingly, a recent study found high levels of ASIC1a in reactive astrocytes in the hippocampi of patients with TLE and epileptic mice. Moreover, selectively inhibiting the expression of ASIC1a on astrocytes by injecting rAAV‐ASIC1a‐shRNA into the dentate gyrus reduced the spontaneous seizures following pilocarpine injection. This finding points to the possible trafficking of ASIC1a in astrocytes during chronic epilepsy pathology.^18^ However, this study did not explore the role of ASIC1a on neurons in the development of epilepsy. Notably, psalmotoxin 1 (PcTX1) effectively and specifically inhibits the ASIC1a current without affecting the currents mediated by other configurations of ASICs, indicating that PcTX1 could be considered an indispensable pharmacological tool for the studies of ASIC1a.^19^ Xiong et al. reported that PcTX1 targeting ASIC1a on neurons protected the brain from ischemic injury by reducing the toxicity of [Ca^2+^]_i_.^20^ However, whether PcTX1 could exert neuroprotective effects and be a potential direction for DMTs in epilepsy remains unknown.

Ferroptosis is an emerging form of programmed cell death mainly associated with three factors: abnormal iron metabolism, depletion of reduced glutathione (GSH)/glutathione peroxidase 4 (GPx4)/cystine‐glutamate transporter protein system (system Xc‐), and abnormal lipid peroxidation.^21^, ^22^, ^23^ When intracellular iron is overloaded, the excess iron generates reactive oxygen species (ROS) via the Fenton reaction, which promotes lipid peroxidation.^24^ In doing so, the glutathione antioxidant system is weakened, contributing to subsequent cell death.^25^ Several studies have demonstrated that ferroptosis plays a prominent role in multiple neurological diseases such as Huntington's disease, stroke, traumatic brain injury, and epilepsy.^26^, ^27^, ^28^ Notably, abnormal iron metabolism is associated with epilepsy. Cortical iron injections can induce recurrent seizures, which are used as a model of post‐traumatic epilepsy.^29^ Moreover, a previous study verified that inhibiting ferroptosis could mitigate pentylenetetrazol kindling, and pilocarpine‐induced seizures in mice, suggesting that ferroptosis‐mediated pathological alterations play important roles in the initiation and progression of epilepsy.^30^, ^31^ However, few studies evaluate the neuroprotective effects of suppressing ferroptosis in epilepsy. [Ca^2+^]_i_ plays an important role in regulating neuron function under physiological and pathophysiological conditions, such as cell death. Importantly, when ASIC1a opens, Ca^2+^ enters in.^32^ However, whether blocking ASIC1a could exert neuroprotective effects through rescuing the ferroptosis associated with [Ca^2+^]_i_ following epilepsy remains unclear.

In this study, we first aimed to confirm the ferroptosis phenomenon on both brain tissues and blood samples in adult patients with epilepsy. Then, we investigated whether ASIC1a inhibitors‐PcTX1 could exert neuroprotective effects using an in vitro epilepsy model and its associated underlying mechanisms to probe its potential value as a DMT for epilepsy.

## 2 METERIALS AND METHODS

### 2.1 Chemicals and reagents

Dulbecco's modified Eagle's medium (DMEM, 11995‐065), neurobasal medium (21103‐049), fetal bovine serum (FBS, 12483‐020), B27 supplement (17504‐044), and penicillin–streptomycin (PS, 15140‐122) were purchased from GIBCO (Grand Island, NY, USA). The ASIC1a inhibitor Psalmotoxin‐1 was obtained from Abcam (ab120483, Cambridge, UK). The ferroptosis inhibitor ferrostatin‐1 was purchased from MCE (HY‐100579, MCE, China).

### 2.2 Brain samples collection

**FIGURE 1:**
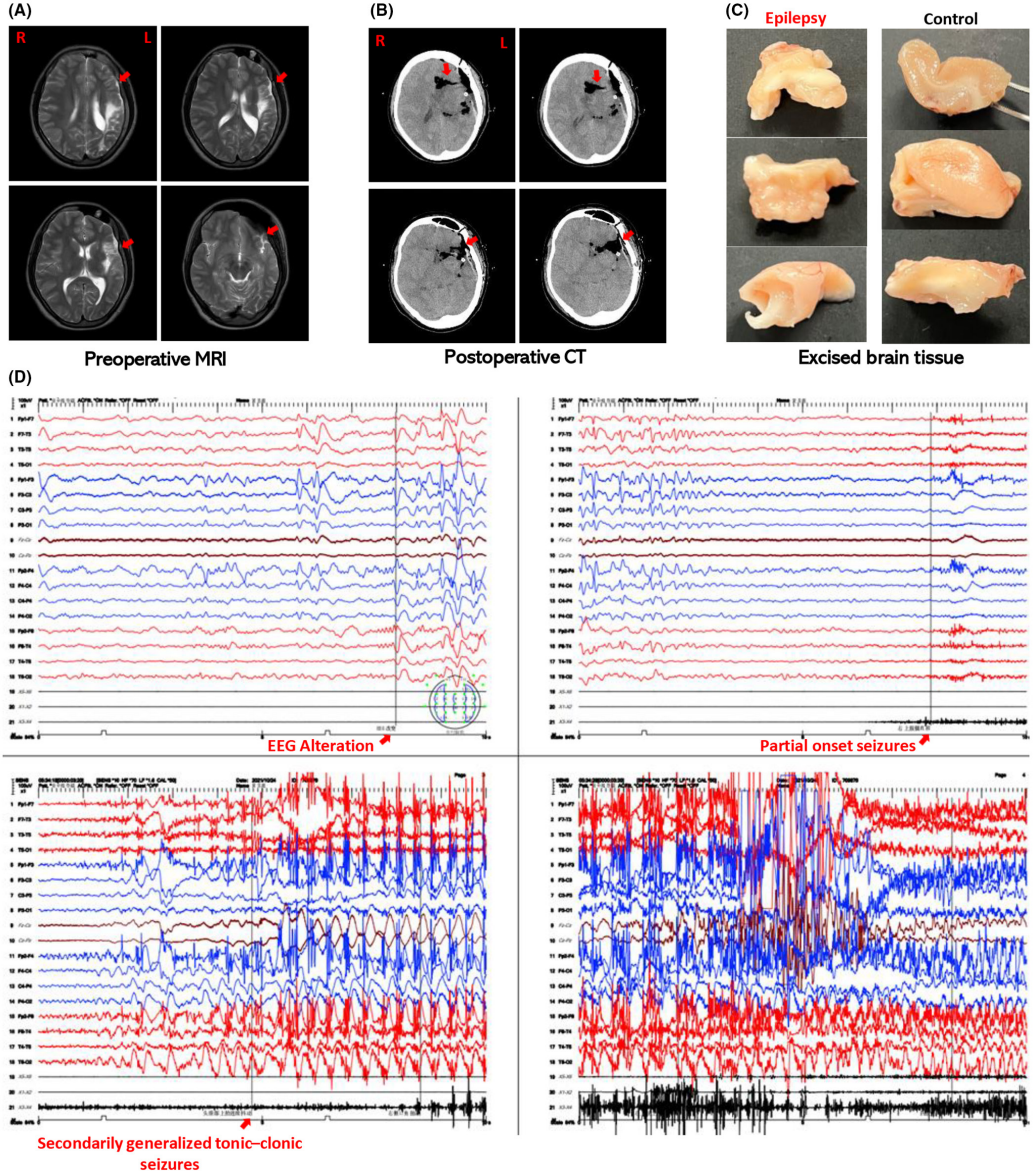
Selection of the brain tissue for epilepsy and control groups. Representative presurgical evaluation of a patient was presented (A, D). (A) The preoperative magnetic resonance imaging (MRI): abnormal parasagittal T2‐weighted MRI of the left frontotemporal regions, which is considered to be the epileptogenic zone; (B) the postoperative computed tomography (CT): CT after brain tissue resection; (C) all resected tissues were classified into two groups, namely, epilepsy and control groups; (D) the scalp video‐EEG monitoring of ictal states of focal seizures evolves to generalized seizures: 3–5 s spike and wave discharges in left anterior regions displayed prior to 3–5 s diffuse low voltage and then spike and wave discharges in the bilateral frontotemporal areas rapidly generalized to all leads.

Brain tissues used in this study were acutely resected from 5 patients with DRE who underwent surgical treatment. All patients were diagnosed with DRE based on the ILAE updated definition and were hospitalized for preoperative evaluation at the Epilepsy Center in the Beijing Tiantan Hospital of Capital Medical University.^3^ We selected the neocortical regions in the epileptogenic zone and corresponding distant non‐epileptic temporal neocortex based on preoperative evaluation, including clinical history, neurological examination, scalp video‐electroencephalogram (EEG) monitoring, magnetic resonance imaging (MRI), and 18‐fluoro‐deoxyglucose positron emission tomography, as epilepsy and control groups, respectively.^33^, ^34^ All resected tissues were presented by using photographs taken after surgery and classified into two groups, namely, epilepsy and control groups (Figure 1C). Compared with the preoperative MRI, the postoperative CT clearly showed the location and extent of the resected zone (Figure 1A,B). Electroencephalography showed the typical epileptic pattern of activities in the frontotemporal region, which represented the resected epileptogenic zone for the epilepsy group (Figure 1D). Here, to safeguard brain tissue function and tissue activity, we collected only two tissues in the resected regions as epilepsy and control group from the same patient, which was approved by the surgery scheme. All human tissues were obtained with patient consent. This study was approved by the Medical Ethics Committee of Tiantan Hospital, Capital Medical University (Beijing, China).

### 2.3 Blood sample collection

We enrolled 13 unrelated patients diagnosed with epilepsy and 15 age‐matched healthy controls (Table 1). The age‐matched healthy adults were collected at the health examination center without a diagnosis of any neurological diseases in the Beijing Tiantan Hospital of Capital Medical University for routine peripheral blood tests. Considering the effect of multiple factors on ferroptosis, there were exclusion criteria to reduce bias, such as intake of antioxidants in 6 months and intake of iron products in 1 year. All participants signed an informed consent, and the study was approved by the Medical Ethics Committee of Tiantan Hospital, Capital Medical University (Beijing, China).

**TABLE 1:** Demographic and clinical data of adults with epilepsy.

Case	Gender	Age (Years)	Course (Years)	Sz frequency (Times/month)	Interval (Days)
1	Male	38	25	3	10
2	Male	32	32	0.25	120
3	Male	27	12	1	60
4	Male	27	4	0.5	60
5	Male	19	3	0.17	60
6	Male	22	18	0.08	180
7	Male	36	34	1	30
8	Male	22	21	4	7
9	Male	20	4	0.33	90
10	Male	25	22	1.5	10
11	Male	49	32	0.42	30
12	Female	19	4	1	21
13	Male	28	11	0.5	30

Peripheral blood samples were collected into EDTA Vacutainer Tubes (Shenzhen, China). After 45 minutes (mins) at room temperature, the plasma was obtained by centrifuging the whole blood for 3 min at 450 ***g***, and it was stored at 80°C for measurements of ferroptosis‐related indices. Take at least 500 μL of whole blood and centrifuge it at 4°C for 5 min. After discarding the supernatant, the precipitate was resuspended in 10‐fold volumes of ice‐cold homogenate, then centrifuged as before and discarded the supernatant. Erythrocytes were lysed by approximately 4 times the volume of ice‐cold Milli‐Q water. Lysate was pelleted by centrifugation at 12,000 ***g*** for 5 min. The supernatant was taken for the determination of GPx enzyme activity.

### 2.4 Cell culture and drug treatments

#### 2.4.1 Primary cortical neuron culture

The protocol of primary neuronal cells was performed using the methods described previously.^35^ Cortices from embryonic rat (E17) were dissected in cold phosphate‐buffered saline. Neural forceps, large scissors, and eye scissors were used to strip embryonic rats, and fine forceps, iris scissors, curved scissors, and eye scissors were used to rotate the brain tissue. The experimental devices were all sterilized in an autoclave. All subsequent operations were performed in a super‐clean table. The tissue was then cut into pieces and digested with 0.05% trypsin and DNAase for 10–20 min. Digestion was terminated by adding DMEM supplemented with 10% fetal bovine serum, 10% horse serum, and 50× penicillin/streptomycin. After filtering the cell suspension with 70‐μm mesh, it was centrifuged at 200 *g*, dispersed, and resuspended with DMEM. Next, cells were mechanically counted and added at densities of 1 × 10^4^, 8 × 10^4^, and 5 × 10^5^ cells/well to poly‐D‐lysine pre‐coated 96‐well, 24‐well, or 6‐well plates, respectively. After 4 h of incubation to allow for cell attachment, DMEM was replaced with NBA Plus [Neurobasal‐A medium supplemented with 1× GlutaMAX, 1× B27 supplement, and 100× penicillin/streptomycin]. Cells were treated with cytarabine (5 μM) and cultured for 3–5 days to inhibit the proliferation of gliocytes. Every 2 days, 50% of the media was replaced with fresh NBA Plus.

#### 2.4.2 Establishment of epilepsy cell model

The in vitro 0‐Mg^2+^ model of epilepsy was established according to previous studies.^36^ After 7–9 days of culture in nutrient medium, cortical neurons were harvested. The nutrient solution was then changed and placed in 0‐Mg^2+^ extracellular medium for 3 hours. This solution contains (mM): 145 NaCl, 2.5 KCl, 2CaCl_2_, 10 HEPES, 10 glucose, and 0.002 glycine (pH = 7.35 and osmolarity maintained at 320 mOsm). This was used as control in normal extracellular (in mM): 140 NaCl, 2.4 KCl, 10 HEPES, 10 glucose, 4 MgCl_2_, and 2 CaCl_2_ (pH = 7.35 and 320 mOsm) for 3 h.

#### 2.4.3 Cell treatment

There are four groups: (1) Control group: The cells were pre‐treated with isodose neurobasal, and then the cells were cultured in normal extracellular culture medium for 3 h. (2) 0‐Mg^2+^ group: The cells were pre‐treated with isodose neurobasal, and then the cells were cultured in 0‐Mg^2+^ extracellular medium for 3 h. (3) PcTX1+ 0‐Mg^2+^ group: The cells were pre‐treated with the concentration gradient of PcTX1 for 1 h, and then the cells were cultured in 0‐Mg^2+^ extracellular medium for 3 h. (4) Fer‐1+ 0‐Mg^2+^ group: The cells were pre‐treated with Fer‐1 for 1 h, and then the cells were cultured in 0‐Mg^2+^ extracellular medium for 3 h. The experimental design was summarized in Figure 4A.

### 2.5 Cell viability assay

Cell viability assays were performed using Cell Counting Kit‐8 (CCK8) (C0038; Beyotime Institute of Biotechnology, China) following the manufacturer's instructions. Briefly, cells were seeded in 96‐well plates in a medium. After drug treatment at the indicated time points, 10 μL CCK8 solution was added to each well and incubated for 3 h. Cell viability was finally measured using a microplate reader at a wavelength of 450 nm.

### 2.6 Electrophysiology

Whole‐cell recordings were performed on primary cortical neurons in parallel on the same day (days 13–16 in vitro), and viewed with an infrared differential interference contrast microscope with an Olympus 40× water immersion lens (BX51WI; Olympus, Tokyo, Japan) at room temperature (22–23°C). Recording electrodes (resistance of 3–6 MΩ) of borosilicate glass capillaries (BF150‐86‐75; Sutter Instruments, Novato, CA, USA) were made by a vertical pipette puller (PC‐100; Narishige, Tokyo, Japan). Resting membrane potential, membrane conductance, and firing properties were determined in current–clamp mode. In current–clamp mode, the pipette solution contained (in mM): 136 K‐gluconate, 17.8 HEPES, 1 EGTA, 0.6 MgCl_2_, 4 ATP, 0.3 GTP, and 12 creatine phosphate (pH 7.4 was adjusted with KOH and osmolarity maintained at 300 mOsm). Standard extracellular solution contained the following (in mM): 140 NaCl, 2.4 KCl, 10 HEPES, 10 glucose, 4 MgCl_2_, and 2 CaCl_2_ (pH = 7.4 and 320 mOsm). The current amplitude of ASIC (I_ASIC_) was recorded under voltage‐mode clamp. The extracellular solution contained (in mM): 150 NaCl, 5 KCl, 1 CaCl_2_, 1 MgCl_2_, 10 HEPES, 10 glucose (pH was adjusted to 7.4 using NaOH and 320 mOsm). Next, 6,7‐dinitroquinoxaline‐2,3‐dione (DNQX, 20 μm) and bicuculline (BIC, 10 μm) were added to the external solution to block the alpha‐amino‐3‐hydroxy‐5‐methyl‐4‐isoxazole propionic acid (AMPA) and GABA currents. Inside the record fluid (in mM): 130 CsCl, 1.6 MgCl_2_, 10 HEPES, 5 EGTA, and Na2‐ATP (pH 7.4 was adjusted with CsOH and osmolarity maintained at 300 mOsm). ASIC currents were elicited by pH drops from 7.4 to 6.0 (Figure 5C). After the voltage clamp mode set at −70 mV was stable for 5 min, the IASIC record was carried out under −80 mV. Data were recorded and acquired at 10 kHz and low‐pass filtered at 1 kHz using a Multiclamp 700B (Molecular Devices, San Jose, CA, USA) and Clampex 10.5 software (Molecular Devices). Clampfit 10.5 was used for offline analysis.

### 2.7 Immunofluorescence staining

Immunofluorescence staining was performed as previously described.^37^ Briefly, frozen sections or cells were incubated with anti‐ASIC1a rabbit polyclonal (1:500, Bioss) and anti‐NeuN mouse monoclonal (1:300, Sigma) antibodies overnight at 4°C, followed by the appropriate secondary antibodies (1:500; Cell Signaling Technology) for 1.5 h at room temperature. Nuclei were counterstained with DAPI for 5 min at room temperature. Confocal images were captured using a laser‐scanning microscope (A1R; Nikon, Tokyo, Japan).

### 2.8 Mitochondria observation by transmission electron microscopy (TEM)

Transmission electron microscopy (TEM) was performed for mitochondrial observation of brain tissue. The prepared tissues were dehydrated and fixed with 2% paraformaldehyde and 2.5% glutaraldehyde. Ultrathin sections of 70 nm thickness were cut and stained with lead citrate and uranyl acetate. Ultrathin sections of the samples were observed and pictured using an electron microscope (H‐7650 system, Hitachi, Chiyoda‐ku, Tokyo, Japan). The ultrastructural changes were assessed empirically by two blinded pathologists from our institute. Mitochondrial ultrastructure from each sample was observed in five random visual fields according to the criteria.

### 2.9 Ferroptosis‐related indicator determination

All samples' proteins were determined by the BCA method (Thermo Fisher, USA).

#### 2.9.1 GPx enzyme activity assay

The GPx enzyme activity in leukocytes, tissue homogenate, and cell extracts was measured by a glutathione peroxidase assay kit (S0056, Beyotime, China). GPx could catalyze GSH to produce glutathione disulfide (GSSG), while glutathione reductase can catalyze GSSG to produce GSH using NADPH, and the level of GPx activity can be calculated by detecting the reduction of NADPH at A340.

#### 2.9.2 Glutathione assay

The GSH levels in the plasma, tissue homogenate, and cell extracts were performed according to the manufacturer's protocols (A006‐1‐1; Nanjing Jiancheng, China). Glutathione levels were detected by using the DTNB‐GSSG reductase recycling methods. A405 was determined by enzyme marker.

#### 2.9.3 Iron assay

The iron concentration in the plasma, tissue homogenate, and cell extracts was measured by using Iron Assay Kit according to the manufacturer's instructions (A039‐2‐1; Nanjing Jiancheng, China). Iron levels were measured by the α, α'‐dipyridyl method and determined by A520.

#### 2.9.4 MDA assay

The MDA concentration in the plasma, tissue homogenate, and cell extracts was determined according to the manufacturer's protocols (A003‐1‐2; Nanjing Jiancheng, China). MDA levels were measured using 2‐thiobarbituric acid methods and followed by determination of A532.

#### 2.9.5 Human 4‐hydroxynonenal ELISA Kit assay

The 4‐hydroxynonenal (4‐HNE) concentration in the plasma was determined according to the manufacturer's protocols (MM‐0796R1; Mlbio, China). The concentration of 4‐HNE in the samples was determined by comparing the O.D.(A450) of the samples to the standard curve.

### 2.10 Mitochondrial membrane potential (ΔΨm)

The mitochondrial membrane potential (ΔΨm) was detected by using the mitochondrial membrane potential assay kit (ab113850; Abcam, Cambridge, MA). The 100 μM carbonyl cyanide 4‐(trifluoromethoxy) phenylhydrazone (FCCP, a potent mitochondrial membrane disruptor)‐treated group was experimentally designed as a positive control group. In brief, after drug treatment, cells were incubated with 100 μL/well fluorescent cationic dye 5,5,6,6′‐tetrachloro‐1,1′,3,3′‐tetraethylbenzimi‐dazoylcarbocyanine iodide (JC‐1) solution at 37°C for 10 min in the dark. Finally, the JC‐1 monomers and JC‐1 aggregates (green fluorescence for monomer, red fluorescence for aggregate) were detected by fluorescent microscopy with exciting light at 490 nm and 535 nm, respectively. Besides, we used a fluorescent microplate reader to record fluorescence at Ex/Em = 490/535 nm.

### 2.11 Measurement of intracellular calcium concentration

Fluorescence imaging and qualification of Ca^2+^ in primary cortical neurons were performed using the indicator dye Fluo‐3AM (S1056; Beyotime Institute of Biotechnology, China). Cells were incubated in Fluo‐3AM (2 μM) for 45 min at 37°C. The Fluo‐3AM‐loaded cells can then be used for qualitative fluorescence imaging and quantitative flow cytometry measurement.

### 2.12 Quantification of ROS in primary cortical neurons

The ROS formation in primary cortical neurons was detected by a cellular ROS assay kit (ab113851; Abcam, Cambridge, MA). According to the manufacturer's instructions, the cellular ROS was detected by utilizing the cell‐permeable reagent 2′,7′‐dichlorofluorescein (DCFDA). After drug treatment, incubate cells with the diluted DCFDA/H2DCFDA and DAPI (100 ng/mL) for 45 min at 37°C in the dark. Finally, the plate was measured immediately on a fluorescence plate reader at Ex/Em = 485/535 nm and observed under a fluorescence microscope.

### 2.13 Western blot analysis

Cell extracts and tissue homogenates were lysed on ice with RIPA buffer (Sigma Aldrich, St. Louis, Missouri, USA), including DTT and protease inhibitors (Protease and Phosphatase Inhibitor Mini Tablets, Thermo Scientific, Waltham, MA, USA). Proteins, in the amount of 30–50 μg, were subjected to SDS PAGE on 4%–12% denaturing gel and probed with the following antibodies: anti‐ASIC1a (1:1000 Abcam, Cambridge, UK), anti‐GPx4 (1:1000, Abcam, Cambridge, UK), and anti‐GAPDH (1:1000, Sigma Aldrich) as a loading control. Horseradish peroxidase‐conjugated goat anti‐mouse or goat anti‐rabbit IgG (1:10,000, Applygen) was used as the secondary antibody, and the signal was visualized using Super ECL Plus substrate (P1050, Applygen). The indicated proteins were quantified with Image J software.

All of the uncropped blots of these western blot experiments were presented in the Data S1.

### 2.14 Statistical analyses

Statistical analyses were done using GraphPad Prism 8 (San Diego, CA, USA) or SPSS 22 (IBM, Armonk, NY, USA). The Shapiro–Wilk test was performed to test for normality. Differences between two independent samples for continuous data were analyzed with Student's *t*‐test or the Mann–Whitney *U* test according to the normality test. Statistical analysis of the cell experiments data was performed by one‐way analysis of variance, respectively, which was followed by a post hoc Dunnett's test. Correlation was determined using Spearman's correlation. *p* < 0.05 was considered statistically significant. Data are expressed as the mean ± SEM.

## 3 RESULTS

### 3.1 Patient cohorts and clinical information

A total of 13 unrelated patients with epilepsy (12 males, 1 female) and 15 age‐matched healthy controls (13 males, 2 females) were enrolled. Detailed clinical information and pathological characteristics of all patients with epilepsy are summarized in Table 1. The patients in the epilepsy group ranged from 19 to 49 years old (median: 27 years old), while that of the control group ranged from 18 to 53 years old (median: 31 years old). As shown in Figure 3E, the mean disease duration was 17.07 years (range 3–34 years), the mean generalized tonic–clonic seizure frequency was 1.06 times/month (range 0.08–4.0 times/month), and the mean interval time between the most recent episode and blood draw was 54.46 days (range 7–180 days). Although a gender bias was present in our study population, it showed no statistical difference in gender distribution between the epilepsy group and the control group.

### 3.2 Increased expression of ASIC1a and ferroptosis occurrence in epileptogenic foci from patients with DRE

**FIGURE 2:**
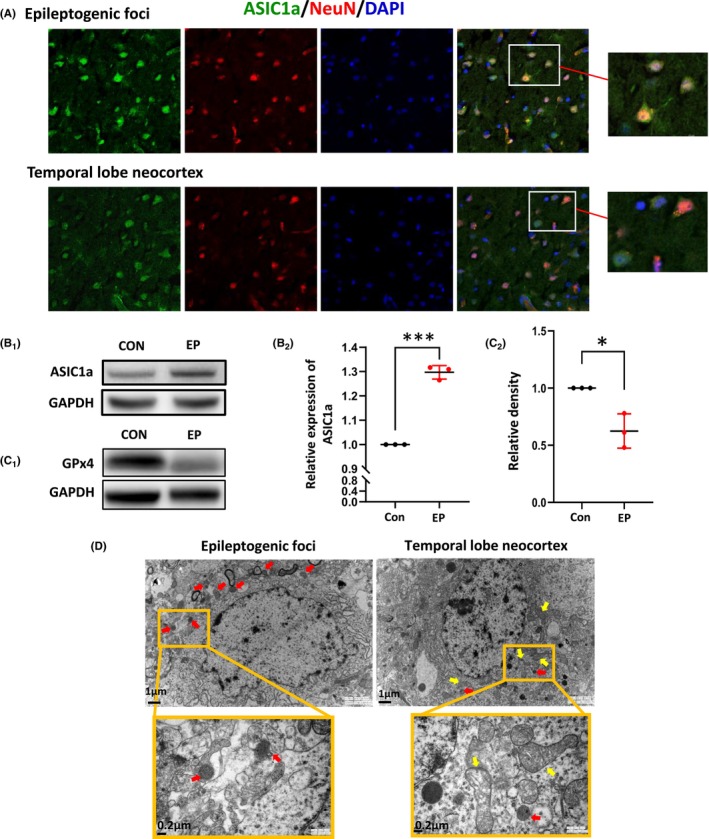
Increased expression of ASIC1a and ferroptosis occurrence in epileptogenic foci from patients with drug‐resistant epilepsy (DRE). (A) Representative confocal photomicrographs of DRE patients with NeuN (red), ASIC1a+ (green), and DAPI (blue) triple‐labeled (scale bar, 10 μm). (B) Representative Western blot images showing brain tissue of ASIC1a (B_1_). Western blot analysis of ASIC1a protein expression in brain tissues (B_2_, *n* = 3 per group). (C) Western blot analysis of GPx4 protein expression in brain tissues (*n* = 3 per group). GAPDH served as a loading control. (D) Mitochondria observation by TEM analysis (scale bar, 200 nm). Yellow arrows, normal mitochondria; Red arrows, crinkled mitochondria. All data are presented as the mean ± SEM. Independent samples *t*‐test was applied for statistical analysis. *p < 0.05, **p < 0.01.

To determine the expression of ASIC1a protein in the DRE, we co‐labeled the ASIC1a with NeuN which is specific to nuclei and perinuclear cytoplasm of most of the neurons in the central nervous system. We found that ASIC1a was expressed on the neurons in the epileptogenic foci (Figure 2A). In addition, we quantified the total expression of ASIC1a and found the level of ASIC1a was higher in the epilepsy group than in the control group (control: 1, epilepsy: 1.30 ± 0.02, *p* < 0.001, Figure 2B). We observed mitochondrial microstructure using electron microscopic to further confirm the occurrence of ferroptosis in DRE. Likewise, we explored the level of GPx4, a crucial mediator of ferroptosis, using Western blotting on brain tissues from epilepsy and control groups. Our results revealed significantly crinkled mitochondria in the epileptogenic foci compared to the relative non‐epileptic neocortex (Figure 2C). In contrast to the control group, there was a significant decrease in the expression of GPx4 in the epilepsy group (control: 1, epilepsy: 0.62 ± 0.09, *p* < 0.05, Figure 2D). To sum up, as far as we know, these results provided direct evidence of the increased level of ASIC1a and ferroptosis in epileptogenic foci from patients with DRE for the first time.

### 3.3 Changes in ferroptosis‐related indices in adult patients with epilepsy

**FIGURE 3:**
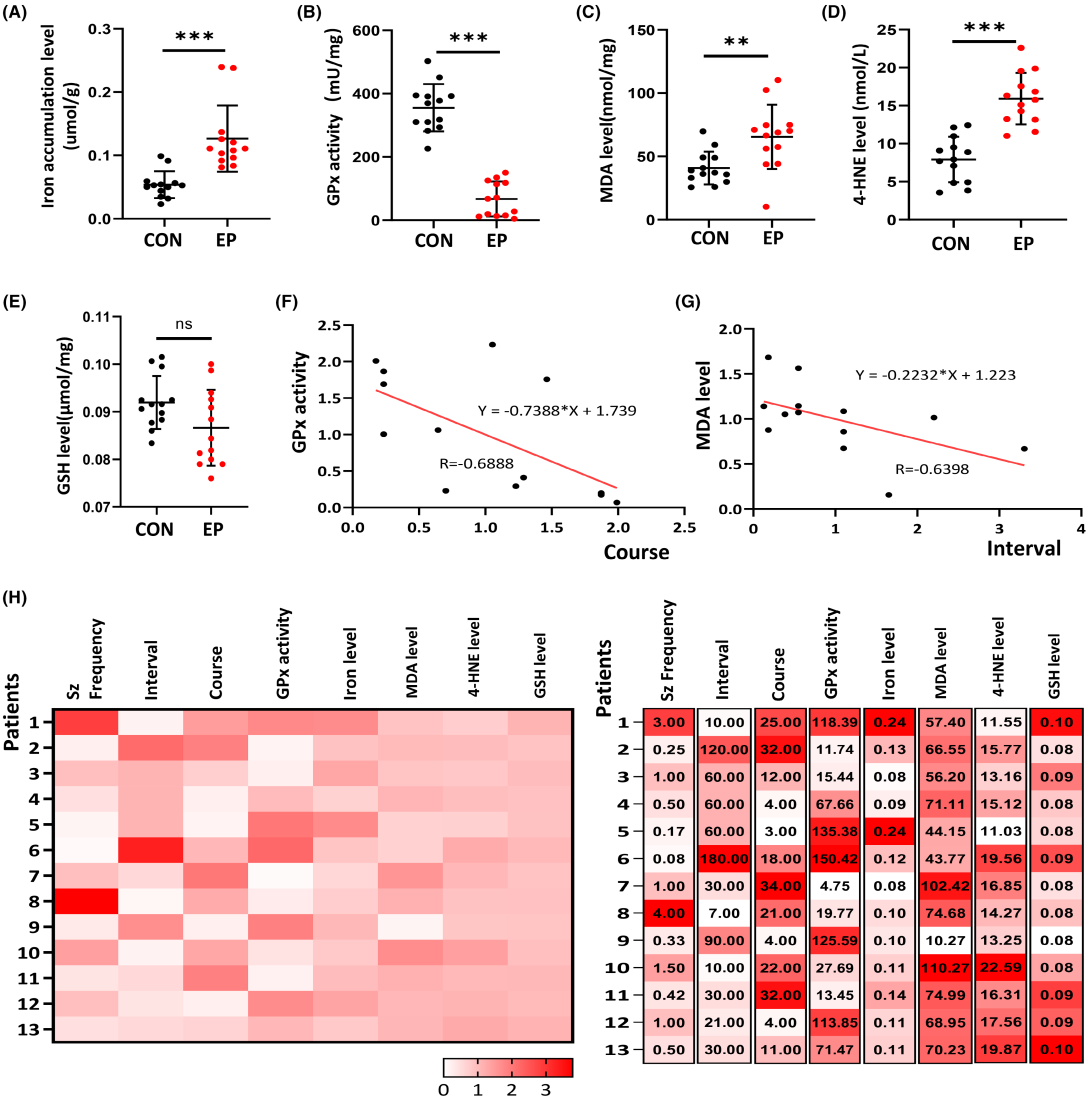
Correlation of clinical information and ferroptosis‐related indices in adult patients with epilepsy. The ferroptosis‐related indices in adult patients with epilepsy (*n* = 13) were analyzed and compared with healthy controls (*n* = 15). (A) Iron accumulation level; (B) GPx activity; (C) MDA concentration; (D) 4‐HNE level; (E) GSH level; the absolute (H, the left side) and relative (H, the right side) values of enrolled patients with epilepsy were listed (*n* = 13). (F) The correlation between GPx enzyme activity and course. (G) The correlation between MDA level and the time interval since the most recent episode. All data are presented as the mean ± SEM. Independent samples *t*‐test was applied for statistical analysis. A correlation was determined using Spearman's correlation. *p < 0.05, **p < 0.01, ***p < 0.001.

A previous study by Petrillo et al. reported the dysregulation of three key ferroptosis markers in children with epilepsy: a consistent increase of 4‐hydroxy‐2‐nonenal (4‐HNE), a significant decrease in glutathione (GSH) levels, and a partial inactivation of the enzyme glutathione peroxidase 4 (GPx4).^38^ Here, we collected peripheral blood samples from adult patients with epilepsy to verify the presence of ferroptosis in adults with epilepsy. Five ferroptosis‐related indices were analyzed: malondialdehyde (MDA) and 4‐HNE, two by‐products of lipid peroxidation; GSH, the primary antioxidant regarded as a direct ROS scavenger in cells; GPx, a critical enzyme in ferroptosis; and plasma iron content.^21^ Compared with the control group, the levels of iron accumulation (control: 0.052 (0.0394, 0.0579) μmol/g, epilepsy: 0.1109 (0.09415, 0.1312) μmol/g, *Z* = −0.426, *p* < 0.001, Figure 3A), MDA **(**control: 40.84 ± 3.59 nmol/mg, epilepsy: 65.46 ± 7.04 nmol/mg, *p* < 0.01, Figure 3C), and 4‐HNE (control: 7.92 ± 0.83 μmol/L, epilepsy: 15.91 ± 0.94 μmol/L, *p* < 0.001, Figure 3D) in the blood increased in adult patients with epilepsy. The GPx enzyme activity in leukocytes was significantly decreased in the epilepsy group **(**control: 355.40 ± 20.72 mU/mg, epilepsy: 137.3 ± 20.16 mU/mg, *p* < 0.001, Figure 3B). However, there was no significant difference in the level of GSH between epilepsy and control groups (Figure 3E). These results powerfully articulated the presence of ferroptosis in adult patients with epilepsy.

Next, we measured the patient's clinical characteristics, including seizure frequency (Sz frequency), the interval time between the most recent episode and blood draw and course, to check the relationship between clinical symptoms and the ferroptosis‐related indices. We listed the absolute and relative values of enrolled patients with epilepsy (Figure 3H). Interestingly, it showed a negative correlation between GPx enzyme activity and course, which meant that the longer course, the significantly lower GPx enzyme activity was found in epilepsy (*R*: −0.69, 95% CI: −0.89 to −0.15, *p* = 0.01, Figure 3F). In addition, a negative correlation was found between the MDA level and the time interval since the most recent episode, meaning that the closer the last seizure attack, the higher level of MDA was found in the plasma sample (*R*: −0.64, 95% CI: −0.88 to −0.12, *p* = 0.02, Figure 3G). Therefore, these suggested that ferroptosis was an irreplaceable part of the pathological alterations in epilepsy.

### 3.4 PcTX1 recured the cell death induced by 0‐Mg^2+^

**FIGURE 4:**
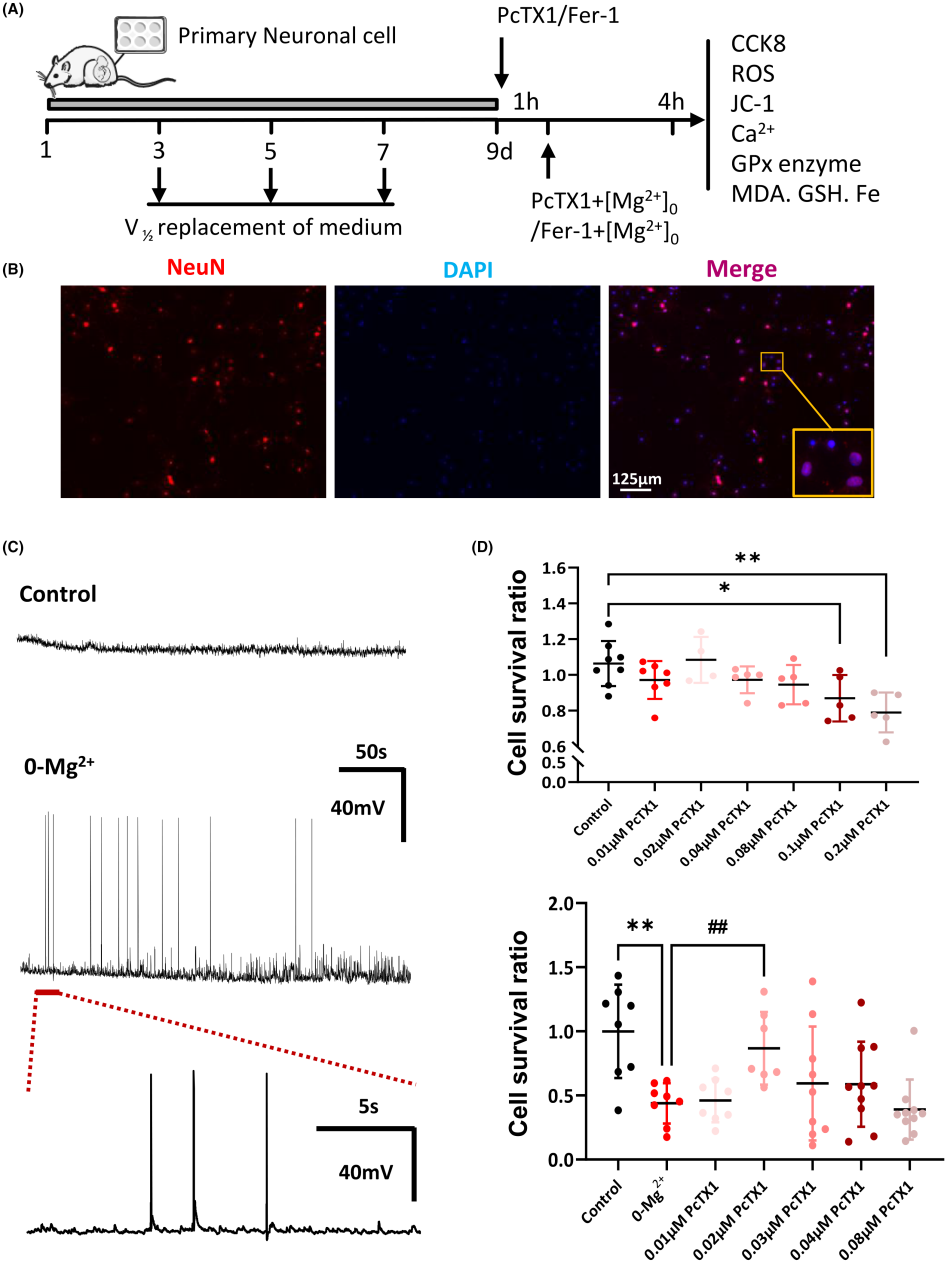
Experimental design and PcTX1 recured the cell death induced by 0‐Mg^2+^. (A) Schematic of experimental timeline in vitro. (B) Representative immunofluorescence staining of NeuN in primary cortical neurons (scale bar, 125 μm, *n* = 3 fields per slide). (C) The electrophysiology studies on the cultured primary cortical neurons, induced by the 0‐Mg^2+^ extracellular medium (*n* = 4). (D, the upper row) CCK8 assessed the cell viability 24 h after PcTX1 (0.01, 0.02, 0.04, 0.08, 0.10, 0.20 μM) treatment (*n* = 4–8 per group). (D, the under row) After being pretreated with PcTX1 (0.01, 0.02, 0.04, 0.08 μM) for 1 h, primary cortical neurons were exposed to both PcTX1 and 0‐Mg^2+^ for 3 h (*n* = 7–10 per group). All data are presented as the mean ± SEM. One‐way analysis of variance was performed for statistical analysis. *p < 0.05, **p < 0.01 versus control group; ^##^ *p* < 0.01 versus 0‐Mg^2+^ group.

To further explore the potential value of PcTX1, a specific blocker of ASIC1a in DMTs, we established a 0‐Mg^2+^‐induced epilepsy model on primary cortical neuronal to mimic the epileptic state reported in a previous study.^36^ For identification of the primary cortical neurons in culture, the primary cultured cortical neurons were immunostained for DAPI and NeuN (Figure 4B). The purification of primary cortical neurons is 93.75%. Electrophysiology studies revealed that the 0‐Mg^2+^ extracellular medium‐induced recurrent spontaneous seizure‐like activities on the cultured primary cortical neurons (Figure 4C). Next, we evaluated the safety of the PcTX1 and examined its effects on rescuing cellular survival rate using CCK8 analyses (Figure 4D). As expected, it only showed significant inhibition on the cellular survival rate with the relatively high concentration of PcTX1 (control: 1.06 ± 0.04; vs. PcTX1 with 0.1 μM: 0.87 ± 0.06, *p* < 0.05; PcTX1 with 0.2 μM: 0.79 ± 0.05, *p* < 0.01, Figure 4D, the upper row), which indicated the significant cytotoxicity on primary cortical neurons. However, the results from the CCK8 assay showed that treatment with 0‐Mg^2+^extracellular medium for 3 hours significantly reduced the survival rate of primary cortical neurons (control: 1.00 ± 0.13, vs. 0‐Mg^2+^: 0.44 ± 0.06, *p* < 0.01, Figure 4D, the under row). To explore the potential effects of pretreatment with PcTX1 on 0‐Mg^2+^‐induced cellular injury, primary cortical neurons were exposed to different concentrations of PcTX1 (0.01, 0.02, 0.04, and 0.08 μM) for 1 h. Notably, they demonstrated that PcTX1 exerted the most apparent protective effects with a concentration of 0.02 μM (PcTX1 with 0.02 μM: 0.87 ± 0.11, *p* < 0.5, Figure 4D, the under row).

### 3.5 PcTX1 inhibited the increased ASIC1a on the in vitro 0‐Mg^2+^‐induced epilepsy model

**FIGURE 5:**
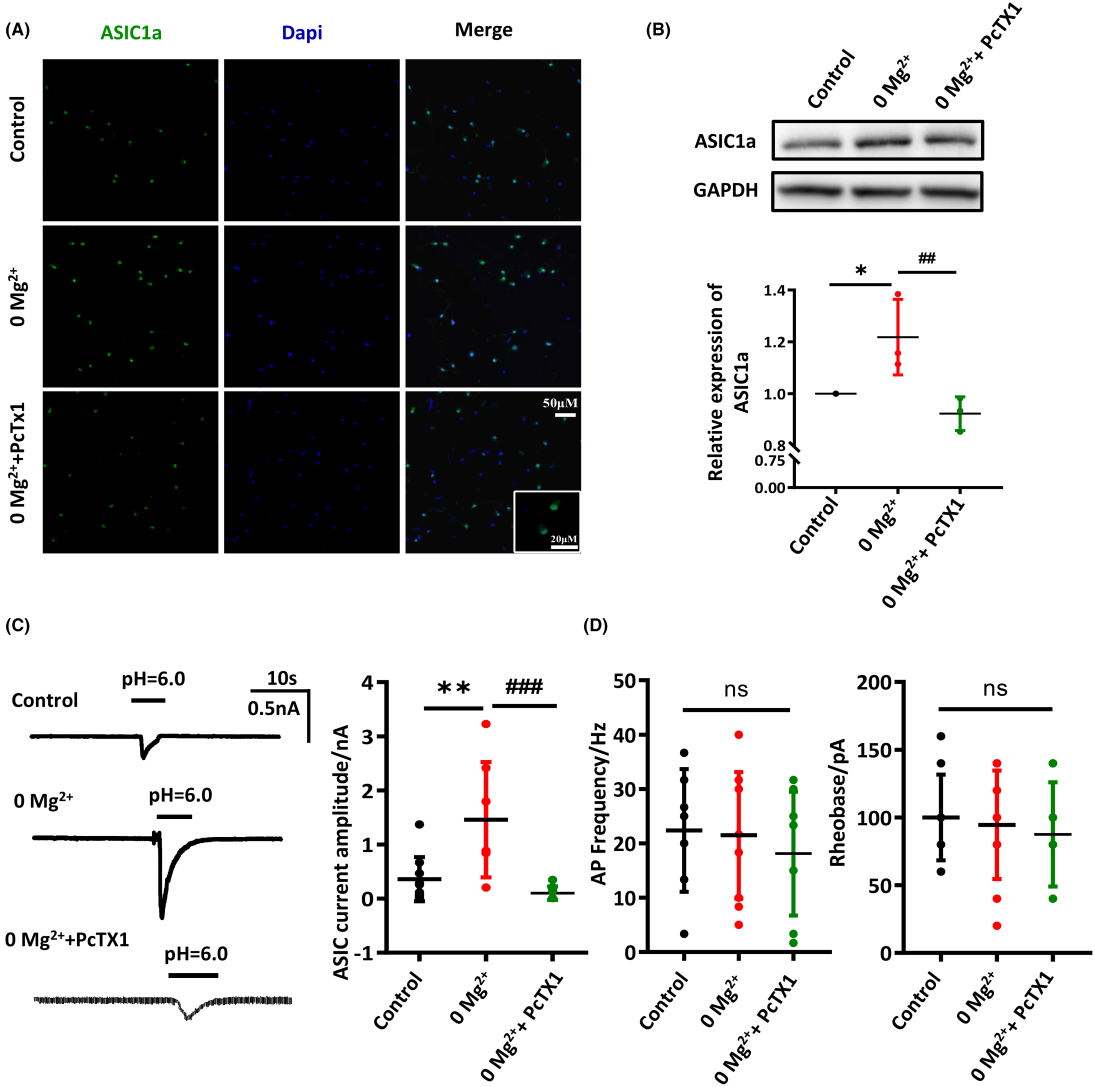
PcTX1 inhibited the increased ASIC1a on the in vitro 0‐Mg^2+^‐induced epilepsy model. The primary cortical neurons were pre‐treated with 0.02 μM PcTX1 for 1 h and 0‐Mg^2+^ treatment for 3 h. (A) Representative immunofluorescence staining of ASIC1a in primary cortical neurons (scale bar, 50 μm, *n* = 3 times per group). (B) Western blot analysis of ASIC1a protein expression in primary cortical neurons (*n* = 3 times per group). (C) The electrophysiological analysis of the ASIC1a currents in primary cortical neurons (*n* = 7–10 per group). (D) The analysis of action potential frequency and rheobase in primary cortical neurons (*n* = 7–11 per group). All data are presented as the mean ± SEM. One‐way analysis of variance was performed for statistical analysis. ns, no significance; *p < 0.05, **p < 0.01 versus control group; ^##^ *p* < 0.01, ^###^ *p* < 0.001 versus 0‐Mg^2+^ group.

We next confirmed the increased ASIC1a in the 0‐Mg^2+^ group, and that PcTX1 could recover the changes of ASIC1a following 0‐Mg^2+^ induction in the subsequent experiments. As expected, we found an increased fluorescence intensity of ASIC1a in the 0‐Mg^2+^ group. Moreover, we observed the reduced fluorescence intensity of ASIC1a in neurons pre‐treated and treated with PcTX1 compared with the 0‐Mg^2+^ group (Figure 5A). Consistent with immunohistochemistry staining, Western blot showed significantly increased expression of ASIC1a in the 0‐Mg^2+^ group, while PcTX1 pretreatment significantly reversed the increase in ASIC1a (control: 1, 0‐Mg^2+^ group: 1.22 ± 0.08, PcTX1 + 0‐Mg^2+^ group: 0.92 ± 0.04, *p* < 0.01, Figure 5B). As reported in the previous study, ASIC1a responded to the pH for half‐maximal activation (pH_50_) at 6.2, mediating fast decaying and transient currents.^11^ Thus, extracellular solution adjusted to pH = 6.0 was applied by a hand‐made drug delivery device which could achieve rapid perfusion within 10 s to the cultured cortical neurons to activate ASIC1a and record the corresponding currents. To know whether 0‐Mg^2+^ could influence acid‐evoked currents, we determined whether pretreatment of cells with 0‐Mg^2+^/PcTX1 affects the amplitude of ASIC currents. Electrophysiological data then revealed that ASIC1a currents (I_ASIC_) were significantly elevated after 0‐Mg^2+^ extracellular medium treatment compared to the control group, while pretreatment with the PcTX1 significantly reversed the elevation of I_ASIC_ (control: 0.36 ± 0.13 nA; vs. 0‐Mg^2+^ group: 1.46 ± 0.40, *p* < 0.01; vs. PcTX1 + 0‐Mg^2+^ group: 0.10 ± 0.04, *p* < 0.001, Figure 5C). However, there were no significant differences in action potential frequency and rheobase (Figure 5D), as well as resting potential (Data S2). Thus, these results indicated that both of expression and function of ASIC1a were enhanced following 0‐Mg^2+^ induction and PcTX1 inhibited the increased ASIC1a in the in vitro 0‐Mg^2+^‐induced epilepsy model.

### 3.6 PcTX1 exerted neuroprotective effects by inhibiting the ferroptosis pathway

**FIGURE 6:**
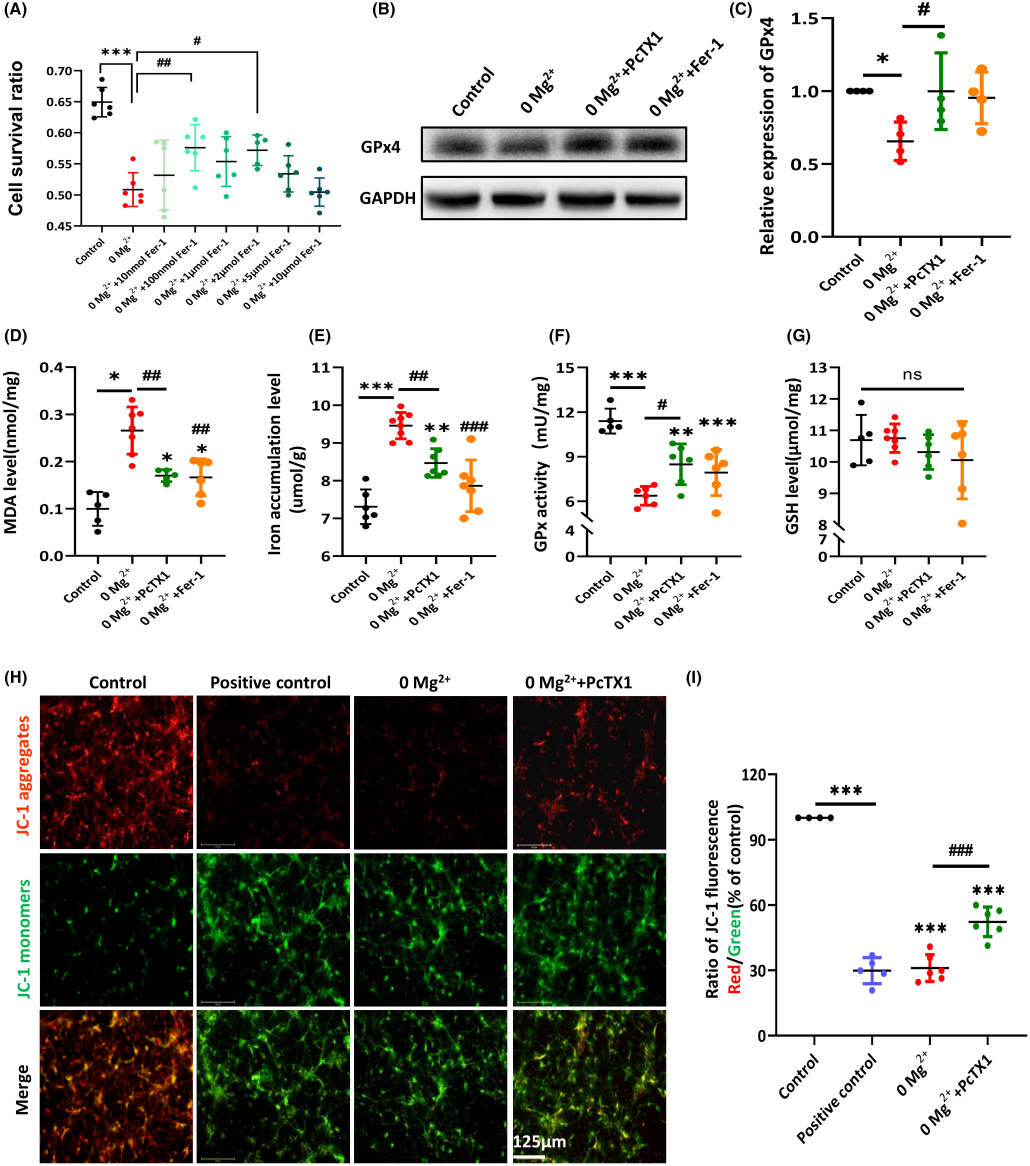
PcTX1 exerted neuroprotective effects by inhibiting the ferroptosis pathway. (A) CCK8 assessed the cell viability exposed to Fer‐1 and 0‐Mg^2+^ for 3‐h after a 1 h Fer‐1 (0.01, 0.1, 1, 2, 5, 10 μM) pre‐exposure (*n* = 5–6 times per group). (B, C) Western blot analysis of GPx4 protein expression in primary cortical neurons (*n* = 4 per group). The ferroptosis‐related indices in primary cortical neurons were analyzed (*n* = 5–8 per group, D–F). (D) MDA concentration; (E) iron accumulation level; (F) GPx activity; (G) GSH level; (H) representative JC‐1 staining pictures of primary cortical neurons (scale bar, 125 μm). JC‐1 monomer emits green light in the cytoplasmic matrix; JC‐1 aggregate in mitochondria emits red light. (I) Ratio of JC‐1 aggregate/JC‐1 monomer was measured for mitochondrial membrane potential (red/green fluorescent area (*n* = 4–6 per group). All data are presented as the mean ± SEM. One‐way analysis of variance was performed for statistical analysis. ns, no significance; *p < 0.05, **p < 0.01, ***p < 0.001 versus control group; ^#^ *p* < 0.05, ^##^ *p* < 0.01, ^###^ *p* < 0.001 versus 0‐Mg^2+^ group.

To explore whether PcTX1 exerted neuroprotective effects through regulating the ferroptosis pathway, we added the Fer‐1, which has been proven to be a ferroptosis inhibitor,^39^ to the following experiments. First, after exposure to a 0‐Mg^2+^ extracellular medium, Fer‐1 showed a similar effect on rescuing cell death. When pre‐treated with Fer‐1 with different concentrations of Fer‐1 (0.01, 0.1, 1, 2, 5, and 10 μM) for 1 h, Fer‐1 exerted the most apparent protective effects with a concentration of 0.1 and 2 μM (control: 0.65 ± 0.01; vs. 0‐Mg^2+^: 0.51 ± 0.01, *p* < 0.001; vs. Fer‐1 with 0.1 μM: 0.58 ± 0.02, *p* < 0.01; vs. Fer‐1 with 2 μM: 0.57 ± 0.01, *p* < 0.05, Figure 6A). Next, when exposed to the 0‐Mg^2+^ solution, it exhibited significantly elevated expression levels of GPx4 (control: 1, vs. 0‐Mg^2+^: 0.66 ± 0.07, *p* < 0.05, Figure 6B,C), MDA (control: 0.10 ± 0.02 nmol/mg, vs. 0‐Mg^2+^: 0.26 ± 0.02 nmol/mg, *p* < 0.001, Figure 6D), and iron contents (control: 7.31 ± 0.19 μmol/g, vs. 0‐Mg^2+^: 9.46 ± 0.12 μmol/g, *p* < 0.001, Figure 6E), decreased GPx enzyme activity (control: 11.39 ± 0.37 mU/mg, vs. 0‐Mg^2+^: 6.37 ± 0.26 mU/mg, *p* < 0.001, Figure 6F), and inequivalent GSH content compared to the control group (Figure 6G). Furthermore, pretreatment with PcTX1 (0.02 μM) reversed the changes above, including the expression level of GPx4 (0‐Mg^2+^+PcTX1: 1.00 ± 0.13, *p* < 0.05; 0‐Mg^2+^+Fer‐1: 0.95 ± 0.09, *p* = 0.07, Figure 6B,C), MDA (vs. 0‐Mg^2+^+PcTX1: 0.17 ± 0.01 nmol/mg, *p* < 0.01; vs. 0‐Mg^2+^+Fer‐1: 0.17 ± 0.02 nmol/mg, *p* < 0.01, Figure 6D), iron (vs. 0‐Mg^2+^+PcTX1:8.45 ± 0.14 μmol/g, *p* < 0.01; vs. 0‐Mg^2+^+Fer‐1: 7.86 ± 0.26 μmol/g, *p* < 0.001, Figure 6E), and GPx enzyme activity (Figure 6F), which exhibited a similar effect with Fer‐1.

The direct pathological histological alteration of ferroptosis is mitochondrial alteration.^21^, ^40^ Consistent with the mitochondrial crumpling found in the brain tissue of patients with DRE, we used the fluorescent cationic dye JC‐1 method to investigate whether PcTX1 mediates the mitochondrial membrane function using the in vitro 0‐Mg^2+^‐induced epilepsy model (Figure 6H). The fluorescence transition from red to green indicated the loss of ΔΨm and hence significant mitochondrial damage. FCCP (a potent mitochondrial membrane disruptor) is used as the positive control group. Similar to positive control (positive control: 29.85 ± 2.67%), there are significant decreases in ΔΨm in the neurons treated with 0‐Mg^2+^ extracellular medium for 3 h when compared with the control group (control: 100%, vs. 0‐Mg^2+^: 31.02 ± 2.51%, *p* < 0.001, Figure 6I). However, PcTX1 could reverse the loss of ΔΨm induced by 0‐Mg^2+^ (0‐Mg^2+^; vs. 0‐Mg^2+^ + PcTX1: 52.27 ± 2.78%, *p* < 0.001, Figure 6I). In summary, these results suggest that the ferroptosis pathway is involved in the 0‐Mg^2+^ solution‐induced epileptic state and PcTX1 could suppress ferroptosis‐related indices and inhibit the ferroptosis like Fer‐1, which indicated that PcTX1 could exert neuroprotective effects in experimental epilepsy.

### 3.7 PcTX1 rebalanced the disputed [Ca^2+^]_i_ homeostasis and reduced oxidative stress reaction induced by 0‐Mg^2+^

**FIGURE 7:**
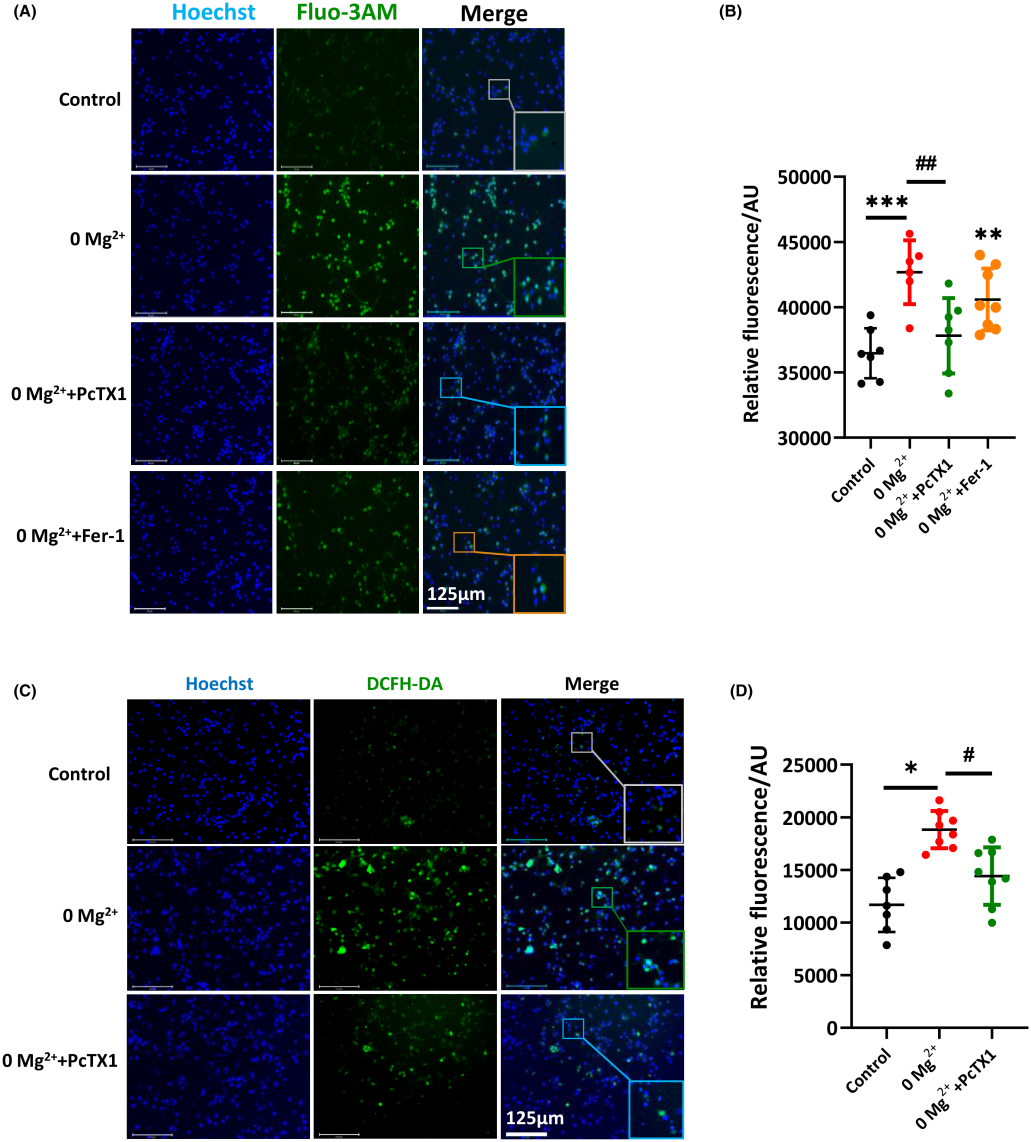
PcTX1 reduced [Ca^2+^]_i_ and oxidative stress reaction induced by 0‐Mg^2+^. (A) The intracellular Ca^2+^ was determined with Fluo‐3 AM. Representative images of the changes in Ca^2+^ were captured (scale bar, 125 μm). (B) Statistical analyses of intracellular Ca^2+^ in different groups were shown (*n* = 6–8 per group). (C) The intracellular ROS were determined with DCFH‐DA. Representative images of the changes of ROS were captured (scale bar, 125 μm). (D) Statistical analyses of intracellular ROS in different groups were shown (*n* = 7–8 per group). All data are presented as the mean ± SEM. One‐way analysis of variance was performed for statistical analysis. ns, no significance; *p < 0.05, **p < 0.01, ***p < 0.001 versus control group; ^#^ *p* < 0.05, ^##^ *p* < 0.01 versus 0‐Mg^2+^ group.

As an indicator of neuronal hyperexcitability, the disputed Ca^2+^ homeostasis, represented as the elevation of Ca^2+^ has been found in numerous studies based on epilepsy models and patients with epilepsy.^41^ Here, we found that intracellular Ca^2+^ was significantly increased after treatment with 0‐Mg^2+^ extracellular medium using Fluo‐3 fluorometry (Figure 7A,B), whereas pretreatment with PcTX1 significantly reversed the increase in the content of [Ca^2+^]_i_ (0‐Mg^2+^: 42677 ± 997.8 AU; vs. 0‐Mg^2+^ + PcTX1: 37812 ± 1090 AU, *p* < 0.01, Figure 7A,B). However, neurons pretreated with Fer‐1 did not change the elevation of [Ca^2+^]_i_ induced by 0‐Mg^2+^ (Figure 7A,B). It was worthy of our attention that PcTX1 was more effective in regulating Ca^2+^ homeostasis than Fer‐1. Given that ferroptosis has been proven to be featured with lipid peroxidation,^42^ here we detected the ROS level to assess the function of PcTX‐1 on intracellular ferroptosis. PcTX1 significantly reduced ROS production in primary cortical neuronal induced by 0‐Mg^2+^ extracellular medium (0‐Mg^2+^: 18826 ± 623.9 AU; vs. 0‐Mg^2+^ + PcTX1: 14406 ± 963.6 AU, *p* < 0.05, Figure 7C,D). These results suggested that PcTX1 could exert neuroprotective effects in experimental epilepsy via inhibiting ferroptosis induced by Ca^2+^ influx.

## 4 DISCUSSION

In this study, we first provided direct evidence of increased ASIC1a and the occurrence of ferroptosis in the brain tissue of patients of DRE. In addition, we found the corresponding changes in ferroptosis‐related indices using blood samples from adult patients with epilepsy compared with age‐matched healthy control. As far as we know, it is the first time to confirm the presence of ferroptosis in the epileptogenic foci from adult patients with DRE and peripheral blood samples from adult patients with epilepsy. The significant findings of our present work demonstrated that PcTX1, a specific blocker of ASIC1a, could rescue neuronal death and exert neuroprotective effects, including reversing the ferroptosis‐related indices, ROS and [Ca^2+^]_i_ alterations on the in vitro 0‐Mg^2+^‐induced epilepsy model. We suggested that inhibiting ASIC1a could play a neuroprotective role on epileptic neurons through attenuating ferroptosis, which implies the potential value of ASIC1a in DMTs for preclinical and clinical studies for epilepsy.

Epilepsy is a chronic neurological disorder, afflicting approximately 50 million people worldwide.^1^ Currently, the first‐line treatments for epilepsy are anti‐seizure medications (ASMs), which are mainly designed for acute seizures control. However, epilepsy is a chronic progressive disease with complicated pathological changes.^5^ Although more than 20 ASMs have been approved for treating epilepsy, no available drug can ameliorate the course of epilepsies and related comorbidities.^6^ In 2002, Löscher et al. first proposed the conception of DMTs and emphasized that the development of DMTs is one of the essential goals for epilepsy in the future.^6^ Our study mainly targeted finding a novel direction for developing DMTs for epilepsy.

It is well known that the oxygen consumption of brain tissue increases, triggering mitochondrial respiratory chain dysfunction and the formation of ROS during seizures due to abnormal hyper‐synchronized discharge.^43^, ^44^, ^45^ Subsequently, oxidative cascade stress and inflammatory reactions occur.^45^, ^46^, ^47^ High expressions of polyunsaturated fatty acids on neuronal membranes make the neurons more sensitive to ROS products.^48^ In addition, releasing excitotoxic substances, such as excessive glutamate and acidic synaptic vesicles, causes tissue acidification, acidotic reactions, and neuron death.^49^, ^50^ Therefore, suppression of excessive oxidative stress and acidosis reactions induced by seizures deserves more attention for protecting neuron function.

ASIC1a, an H^+^‐activated Na^+^ channel sensitive to pH decreasing rapidly with ubiquitous expression in the central nervous system, has been reported to be associated with multiple acidosis‐related diseases.^20^, ^51^, ^52^, ^53^ There existed a difference between rodent and human ASIC1a. It is worth noting that resected cortical tissue from humans exhibits a higher membrane/total ratio of ASIC1a than that from mice.^54^ Channel functions of ASIC1a in different species (human and rat) showed differences in pH sensitivity, the desensitization property, the steady‐state inactivation, and sensitivity to ASIC1a regulators.^54^, ^55^, ^56^ Acidotoxicity mediated by hASIC1a appears to be more severe than that by mASIC1a.^54^ These findings suggest that hASIC1a exhibits a greater response to acid signaling and has a stronger impact on the related biological effects than mASIC1a, so we selected clinically excised samples as experimental subjects. Current studies resolving the correlation between ASIC1a and epilepsy focus on alterations of ASICs protein and the direct effect of its antagonists on acute seizures.^18^, ^57^ In our preliminary study, we found that I_ASIC_ was significantly attenuated when exposed to ketone bodies which have been shown as an effective long‐term treatment for epilepsy, suggesting that ASIC1a may be an essential part of the development and progression of epilepsy.^35^ To further confirm the changes of ASIC1a in epileptogenic foci from patients with chronic epilepsy, we selected inpatients diagnosed with DRE for surgical evaluation as subjects. According to a previous study, we chose the temporal neocortex tissue, which is distant from the epileptogenic zone, from patients with mesial temporal epilepsy as the control group.^34^ Then, we performed immunostaining with anti‐ASIC1a and anti‐NeuN antibodies and observed a high intensity of ASIC1a on the NeuN‐positive cells. We found that the expression of ASIC1a was significantly increased in excised epileptogenic foci compared to the control group. PcTX1, a specific inhibitor of ASIC1a, has been reported to protect the brain from ischemic injury by reducing the toxicity of [Ca^2+^]_i_ by targeting ASIC1a expressed in neurons.^20^ However, there is no study to evaluate whether PcTX1 could exert neuroprotective effects in epilepsy. Undoubtedly, rescuing neuron damage is indeed the core mechanism of anti‐epileptogenesis, and it is also an important breakthrough in the DMTs for epilepsy from a clinical perspective. The acidic extracellular environment can activate ASIC1a to result in a large amount of Na^+^ and Ca^2+^ influx, promoting the release of related neurotransmitters or even causing neuronal damage.^32^, ^49^ 0‐Mg^2+^‐induced primary neuron is a classical epilepsy neuronal damage model: the absence of magnesium removes the block from the NMDA receptor channels and leads to their uncontrolled activation and excessive calcium influx, which can trigger a cascade of events that ultimately affect the activity of AMPA receptors and release excitatory glutamate. The activation of AMPA receptors by glutamate leads to an influx of Na^+^ into the neuron, which further depolarizes the postsynaptic membrane. This depolarization can trigger action potentials and increase the firing rate of the neuron, contributing to neuronal hyperexcitability. Therefore, we chose 0‐Mg^2+^‐induced primary neurons as our in vitro model.^58^, ^59^, ^60^ In our study, PcTX1 was found to rescue 0‐Mg^2+^‐induced neuron death, rebalance the Ca^2+^ homeostasis, and alleviate ROS production induced by 0‐Mg^2+^. However, the detailed mechanism of PcTX1 exerting neuroprotective effects remains unclear.

Ferroptosis is a type of cell death mainly caused by lipid peroxidation of unsaturated fatty acids in the cell membrane.^21^ Emerging evidence suggests that ferroptosis is probably a significant cell death modality in several neurodegenerative pathologies, since iron accumulation, decreased GSH and GPx enzyme levels, and increased lipid peroxidation have been observed in many neurodegenerative processes.^21^, ^22^, ^23^, ^61^, ^62^ Ferroptosis in neurons was also observed in various epileptic animal models, causing neuron death increase, mitochondrial volume reduction, and ferroptosis‐related index changes. After treatment with ferroptosis inhibitor Fer‐1, all the above phenomena could be reversed, and the cognitive function of animals could be improved, suggesting that reversing ferroptosis was an essential component of the neuroprotective effect.^30^, ^31^ Simultaneously, Petrillo et al. found the abnormal expression of ferroptosis indicators in the blood samples of children with epilepsy, which suggests that the determination of ferroptosis indicators may be a biomarker that plays a particular role in evaluating the ferroptosis in the brain tissues and the prognosis of epilepsy.^38^ However, this study was limited to the specific cohort. Therefore, we collected blood from adult patients with epilepsy on the day of hospitalism before the clinical intervention to restore the patient's daily state to the greatest extent. Moreover, we observed the mitochondria and measured the expression of key enzymes of ferroptosis in the epileptogenic foci of patients with DRE. It is the first study to provide direct and indirect evidence to verify ferroptosis in patients with epilepsy, including representative crinkled mitochondria, increased iron deposition, depletion of glutathione, decreased GPx enzyme activity, and increased MDA content. Furthermore, we performed a deeper correlation analysis on changes in ferroptosis indicators in blood with clinical data. We surprisingly found that the activity of GPx, the key enzyme of ferroptosis, was negatively correlated with the duration of the disease. In addition, the content of MDA was negatively correlated with the interval between seizures, suggesting that ferroptosis indicators in blood might become potential biomarkers in DRE. Given that our blood sample collection is limited due to the gender‐biased population and statistical parameters only based on generalized tonic seizures, further research on female patients and different types of DRE needs further improvement. Consistent with the above‐mentioned results, we found similar changes in ferroptosis indicators on the in vitro 0‐Mg^2+^induced epilepsy model.

ASIC1a is the only member of ASICs with specific permeability to Ca^2+^.^20^, ^62^, ^63^ It has been well documented that [Ca^2+^]_i_ overload can provoke cytotoxicity and induce cell death.^64^, ^65^ In a recent study, Minhajuddin et al. found that ferroptosis had been linked to the activation of protein kinase C isoforms, which are Ca^2+^ dependent.^62^ A point worth mentioning is that the crosstalk between Ca^2+^, iron, and ferroptosis is bidirectional via ROS signaling. Likewise, iron‐induced ROS generation elicits RyR‐mediated Ca^2+^ signals that promote ERK1/2 phosphorylation in primary hippocampal cultures kept in a Ca^2+^‐free medium.^66^, ^67^ Besides, Bostanci et al. found that blocking L‐type VGCCs may reduce the neurotoxic effects of iron by inhibiting the cellular influx of excessive Ca^2+^ and/or iron ions.^68^ Notably, excess iron causes mitochondrial fragmentation, elevated Ca^2+^ levels that subsequently stimulate the Ca^2+^‐dependent phosphatase calcineurin, and neuronal cell death in the HT‐22.^69^ Besides, ferroptosis also generates excessive ROS. Consequently, excessive ROS production leads to oxidative stress and is detrimental to neurons. These combined observations support a mechanistic link between iron, Ca^2+^, and ferroptosis in the central nervous system. The balance of the triangle breaks down, the neuronal damage occurs.

**FIGURE 8:**
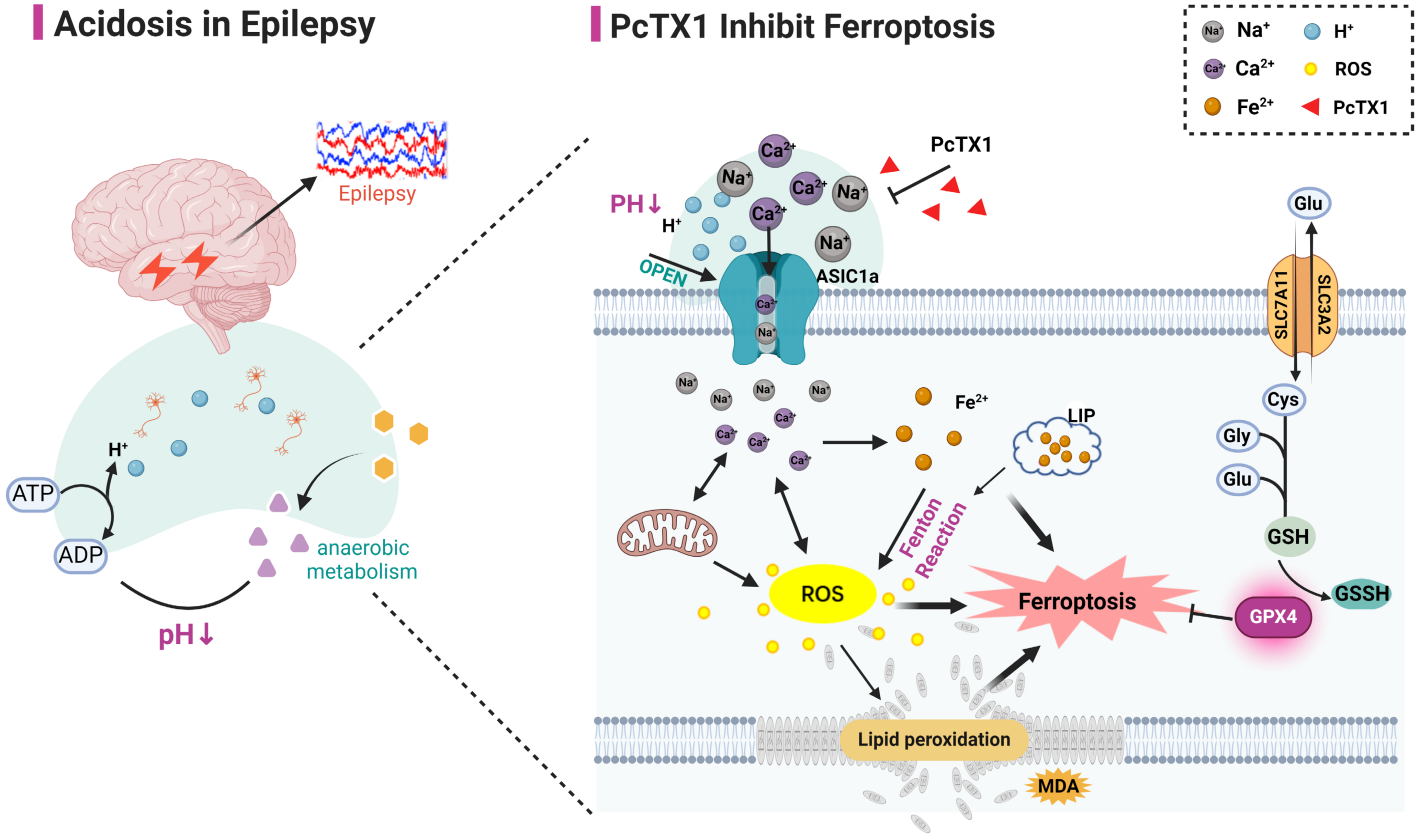
Diagram of the possible mechanism by which PcTX1 regulates the ferroptosis in epilepsy. Left: During the pathogenic process of epilepsy, cellular acidosis developed. Right: ASIC1a opens due to a rapid decrease in pH. The development of epilepsy is accompanied by the occurrence of ferroptosis. PcTX1 exerted neuroprotective effects by inhibiting the ferroptosis pathway.

Therefore, we hypothesize that inhibiting ASIC1a can rescue neuronal death by regulating neuronal ferroptosis via alleviating [Ca^2+^]_i_ overload in epileptic models. Here, we found that PcTX1 significantly recovered the changes of ferroptosis indexes on primary cortical neurons induced by 0‐Mg^2+^, which showed a similar effect as the ferroptosis‐specific inhibitor Fer‐1. In addition, we found that PcTX1 had a stronger inhibition on the Ca^2+^ than Fer‐1. There are two potential reasons to explain it: firstly, ferroptosis might be partially due to Ca^2+^ influx by ASIC1a; second, we proposed the possibility involved in the relationship of the ASIC1a‐Ca^2+^‐ferroptosis axis for epilepsy (Figure 8). In summary, the results suggested that remodeling [Ca^2+^]_i_ might be associated with regulating ASIC1a on ferroptosis.

It is worth mentioning that there still exist some limitations in our research, which are worthy of further research. (1) The mechanism for the change of ASIC1a activity after 0‐Mg^2+^; (2) the difference between human ASIC1a and rat ASIC1a in expression and channel function; and (3) the specific mechanism of [Ca^2+^]_i_ in the ASIC1a‐Ca^2+^‐ferroptosis axis for epilepsy.

## 5 CONCLUSION

In conclusion, our study provided evidence of the increased ASIC1a in epileptogenic foci and ferroptosis in patients with epilepsy. Meanwhile, we suggested inhibiting excessive ASIC1a with PcTX1 could exert neuroprotective effects via mediating neuronal ferroptosis by alleviating [Ca^2+^]_i_ overload. On the one hand, our work reveals a novel strategy of disease‐modifying therapy for epilepsy to prevent ferroptosis‐induced neuronal death, including the regulation of the Ca^2+^ signaling pathway. On the other hand, it further expands the possibility of ASIC1a‐[Ca^2+^]_i_ overload‐ferroptosis in epilepsy, which supports a potential value of ASIC1a as a DMT for epilepsy, providing a theoretical basis for clinical translation.

## AUTHOR CONTRIBUTIONS

Xiaorui Shi and Ru Liu contributed to study design, formal analysis, methodology, visualization, original draft writing. Jianping Wu, Yingting Wang, and Tingting Yu were involved with formal analysis, visualization, review, and editing. Kai Zhang and Chao Zhang provided clinical patient data, tissue specimens, and pathological examination. Yuyu Gu and Limin Zhang collected blood samples from patients. Qun Wang and Fei Zhu conceived the study, conceptualization, data curation, review, and editing, project administration, resources, and supervision of the research.

## FUNDING INFORMATION

This work was supported by the National Key R&D Program of China grant (2022YFC2503800), National Natural Science Foundation of China (8237050142), Beijing Municipal Natural Science Foundation (7232045, 7204256), and CAAE Epilepsy Research Foundation (CJ‐2022‐017).

## CONFLICT OF INTEREST STATEMENT

The authors have no competing interests to declare that could have appeared to influence the work reported in this article.

## Supporting information


Data S1

## Data Availability

Original recordings and images are available upon reasonable request.
