# Supplementary material for: Inhibiting acid‐sensing ion channel exerts neuroprotective effects in experimental epilepsy via suppressing ferroptosis
Source: CNS Neurosci Ther. 2024 Feb 15;30(2):e14596. doi: 10.1111/cns.14596 (PMC10867794; doi:10.1111/cns.14596)

Supplementary 1:Full unedited blot for Figure 2B

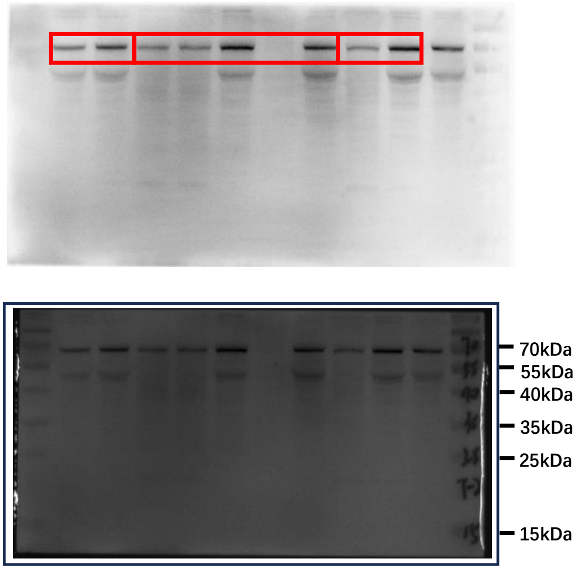

ACCN2

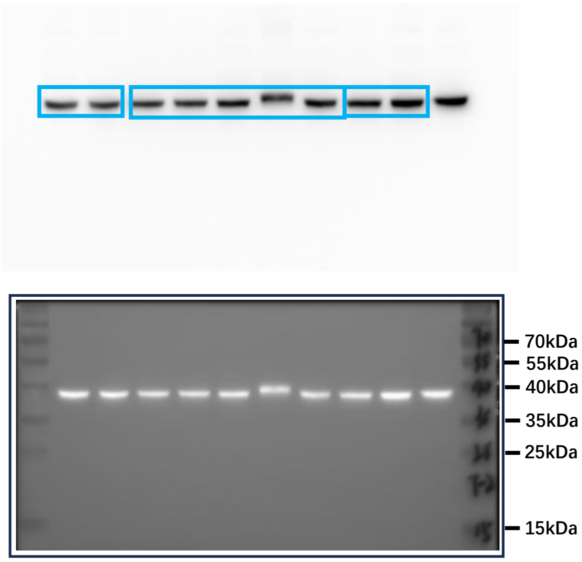

GAPDH

Supplementary 1:Full unedited blot for Figure 2D

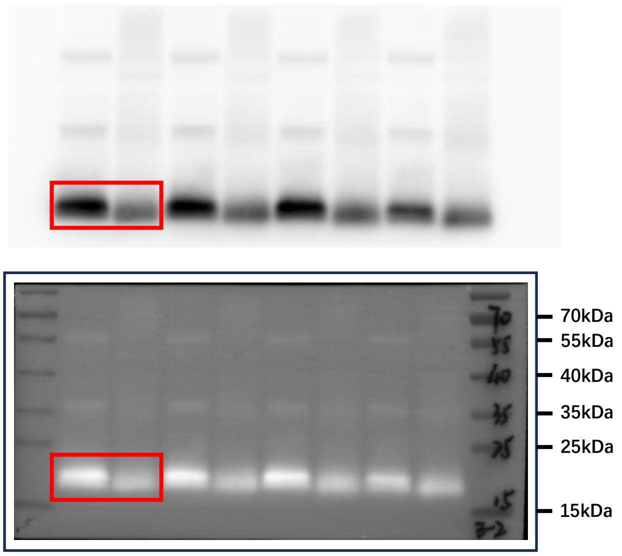

GPX4

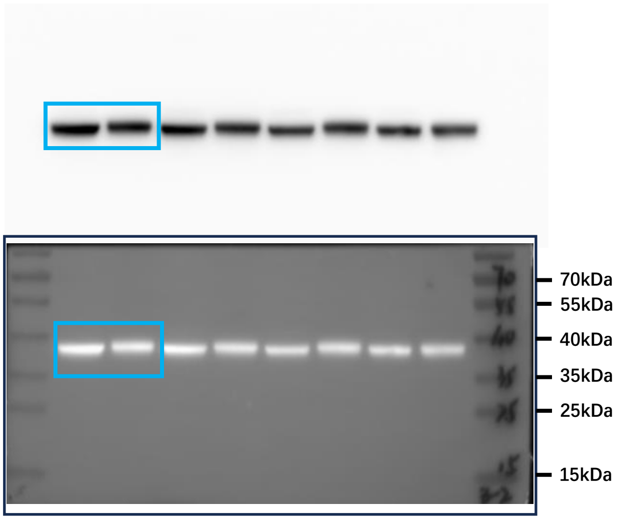

GAPDH

Supplementary 1:Full unedited blot for Figure 5B

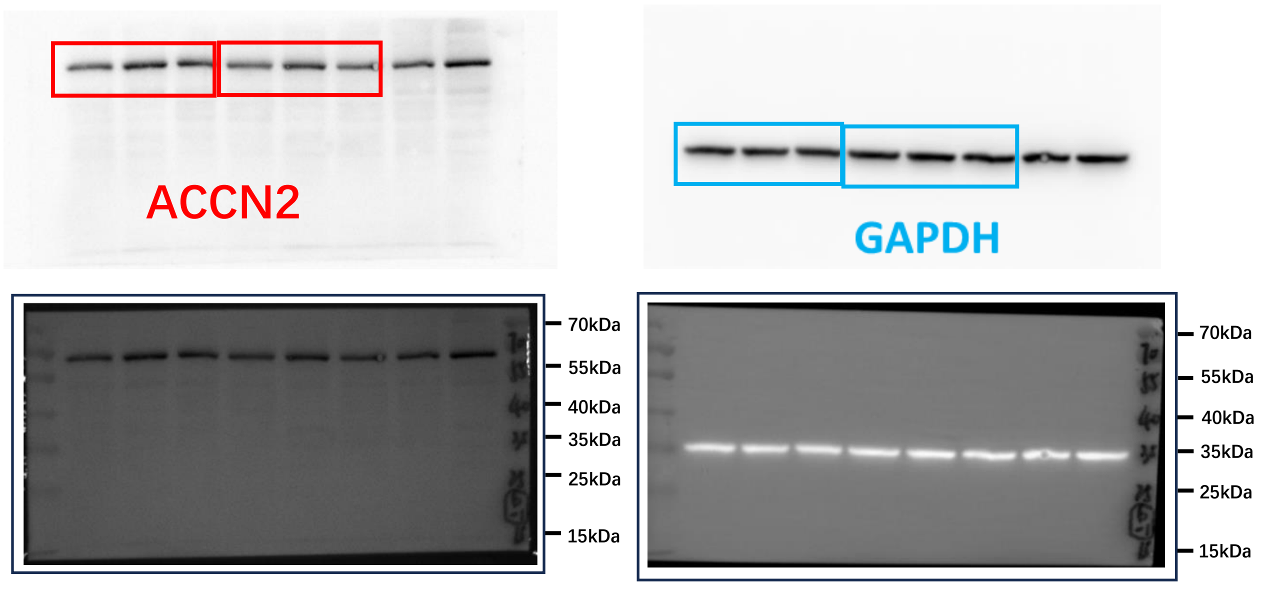

Supplementary 1:Full unedited blot for Figure 6B

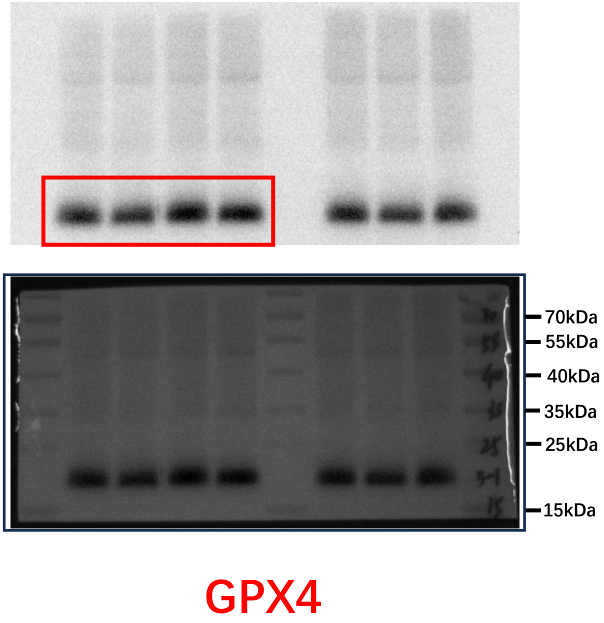

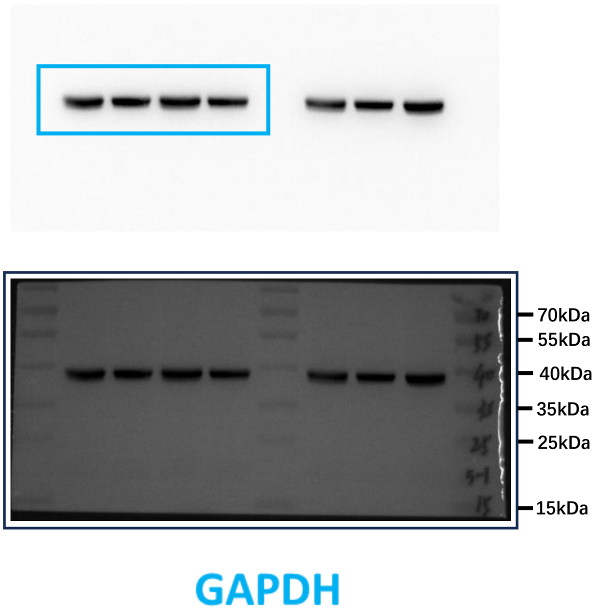

Supplement: Supplementary file 1 — Data S1 [file CNS-30-e14596-s001.zip › DataS1.pdf]
