# Supplementary figures and images for: Inhibiting acid‐sensing ion channel exerts neuroprotective effects in experimental epilepsy via suppressing ferroptosis
Source: CNS Neurosci Ther. 2024 Feb 15;30(2):e14596. doi: 10.1111/cns.14596 (PMC10867794; doi:10.1111/cns.14596)

Supplementary 2: The analysis of resting potential in primary cortical neurons

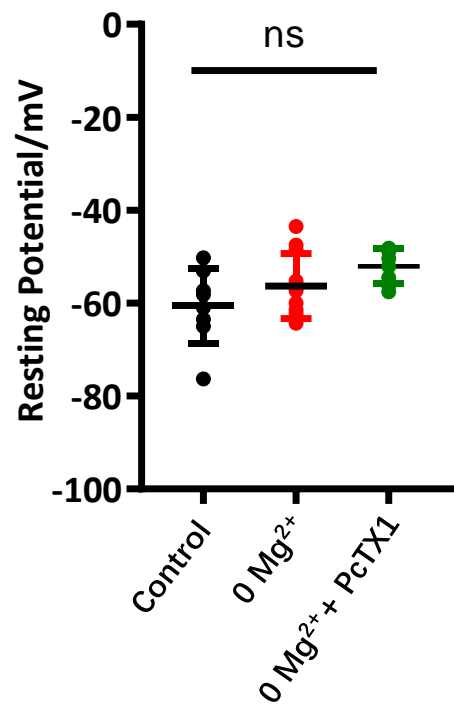

Supplement: Supplementary file 1 — Data S1 [file CNS-30-e14596-s001.zip › DataS2.pdf]
